# The Cellular DNA Helicase ChlR1 Regulates Chromatin and Nuclear Matrix Attachment of the Human Papillomavirus 16 E2 Protein and High-Copy-Number Viral Genome Establishment

**DOI:** 10.1128/JVI.01853-16

**Published:** 2016-12-16

**Authors:** Leanne Harris, Laura McFarlane-Majeed, Karen Campos-León, Sally Roberts, Joanna L. Parish

**Affiliations:** Institute of Cancer and Genomic Sciences, College of Medical and Dental Sciences, University of Birmingham, Birmingham, United Kingdom; International Centre for Genetic Engineering and Biotechnology

**Keywords:** DNA helicase, episome, papillomavirus, persistence, virus-host interaction

## Abstract

In papillomavirus infections, the viral genome is established as a double-stranded DNA episome. To segregate the episomes into daughter cells during mitosis, they are tethered to cellular chromatin by the viral E2 protein. We previously demonstrated that the E2 proteins of diverse papillomavirus types, including bovine papillomavirus (BPV) and human papillomavirus 16 (HPV16), associate with the cellular DNA helicase ChlR1. This virus-host interaction is important for the tethering of BPV E2 to mitotic chromatin and the stable maintenance of BPV episomes. The role of the association between E2 and ChlR1 in the HPV16 life cycle is unresolved. Here we show that an HPV16 E2 Y131A mutant (E2^Y131A^) had significantly reduced binding to ChlR1 but retained transcriptional activation and viral origin-dependent replication functions. Subcellular fractionation of keratinocytes expressing E2^Y131A^ showed a marked change in the localization of the protein. Compared to that of wild-type E2 (E2^WT^), the chromatin-bound pool of E2^Y131A^ was decreased, concomitant with an increase in nuclear matrix-associated protein. Cell cycle synchronization indicated that the shift in subcellular localization of E2^Y131A^ occurred in mid-S phase. A similar alteration between the subcellular pools of the E2^WT^ protein occurred upon ChlR1 silencing. Notably, in an HPV16 life cycle model in primary human keratinocytes, mutant E2^Y131A^ genomes were established as episomes, but at a markedly lower copy number than that of wild-type HPV16 genomes, and they were not maintained upon cell passage. Our studies indicate that ChlR1 is an important regulator of the chromatin association of E2 and of the establishment and maintenance of HPV16 episomes.

**IMPORTANCE** Infections with high-risk human papillomaviruses (HPVs) are a major cause of anogenital and oropharyngeal cancers. During infection, the circular DNA genome of HPV persists within the nucleus, independently of the host cell chromatin. Persistence of infection is a risk factor for cancer development and is partly achieved by the attachment of viral DNA to cellular chromatin during cell division. The HPV E2 protein plays a critical role in this tethering by binding simultaneously to the viral genome and to chromatin during mitosis. We previously showed that the cellular DNA helicase ChlR1 is required for loading of the bovine papillomavirus E2 protein onto chromatin during DNA synthesis. Here we identify a mutation in HPV16 E2 that abrogates interaction with ChlR1, and we show that ChlR1 regulates the chromatin association of HPV16 E2 and that this virus-host interaction is essential for viral episome maintenance.

## INTRODUCTION

Human papillomaviruses (HPVs) are a large family of double-stranded DNA viruses that infect cutaneous and mucosal epithelia, causing hyperproliferative lesions which are at risk of progressing to anogenital, oropharyngeal, and cutaneous cancers. Completion of the HPV life cycle is absolutely dependent on the terminal differentiation of the infected epithelial cells, and during the productive cycle, the ∼8,000-bp viral genome is maintained as extrachromosomal episomes (reviewed in reference 1). Integration of the viral genome into the host genome is considered a risk factor for cancer progression (2). Therefore, the mechanistic underpinnings of viral episome establishment during infection and subsequent episomal maintenance have been the focus of many research studies.

The viral E2 protein plays a complex role in the HPV life cycle. It is essential for viral genome replication, via a well-studied interaction with the viral helicase E1 (3, 4), and for transcriptional regulation of the viral oncogenes E6 and E7 (5, 6). Additionally, E2 is required for the segregation of viral genomes into the nuclear compartment of daughter cells during mitotic cell division, and therefore for episomal maintenance of the viral genomes (reviewed in reference 7). E2-mediated viral genome segregation is dependent on virus-virus and virus-host interactions. The C-terminal DNA-binding domain (DBD) of E2 binds to specific sequences within the viral genome (8), while during mitosis the transactivation domain (TAD) can interact with cellular chromatin by targeting chromatin-bound host cell proteins (9–12), effectively tethering viral episomes to cellular chromatin as infected cells divide. This tethering mechanism is essential for viral episome persistence and productive HPV infection (7).

Several cellular proteins have been suggested as the chromatin-associated mitotic receptor for E2. Interaction with the bromodomain protein Brd4 is essential for bovine papillomavirus 1 (BPV1) E2-mediated viral genome tethering (13) but is not essential for chromosomal attachment of many HPV E2 proteins, including those of the alpha-HPV group (14). It has also been demonstrated that topoisomerase binding protein 1 (TopBP1) has a role in the chromatin association of HPV16 E2 during late stages of mitosis and that interaction with TopBP1 regulates the association of E2 with cellular chromatin (15). Interestingly, a mutant of E2 that is unable to bind TopBP1 was shown to be transcriptionally competent but unable to support virus replication, suggesting that the interaction between E2 and TopBP1 is important for episome establishment (16).

Our previous work identified an interaction between BPV1 E2 and the host cell DNA helicase ChlR1 and showed that ChlR1 interaction was conserved in HPV11 and -16 (17). Following the identification of a mutant BPV1 E2 protein compromised in ChlR1 binding, we demonstrated that interaction with ChlR1 is essential for mitotic attachment of BPV1 E2 and for maintenance of BPV1 genomes (17). However, colocalization studies showed that ChlR1 was not recruited to chromatin-associated E2 foci (17), suggesting that ChlR1 does not function as the mitotic tether for the E2-viral DNA complex but rather plays a role in bringing E2 to the chromatin during DNA replication, prior to mitotic cell division. In support of this hypothesis, characterization of the interaction between BPV1 E2 and ChlR1 by fluorescence resonance energy transfer revealed a more robust association during active DNA replication in S phase of the cell cycle (18).

Following on from these studies, we have now characterized the interaction between HPV16 E2 and ChlR1 and identified a single residue in E2 that is important for interaction with ChlR1, namely, tyrosine 131. Analysis of a ChlR1-binding-defective E2 mutant, E2^Y131A^, provides evidence that interaction with ChlR1 is essential for establishment and maintenance of episomal HPV16 infection and that loss of ChlR1 binding has impacts on E2 function by altering its chromatin association during S phase.

## RESULTS

### HPV16 E2 associates with ChlR1.

**FIG 1:**
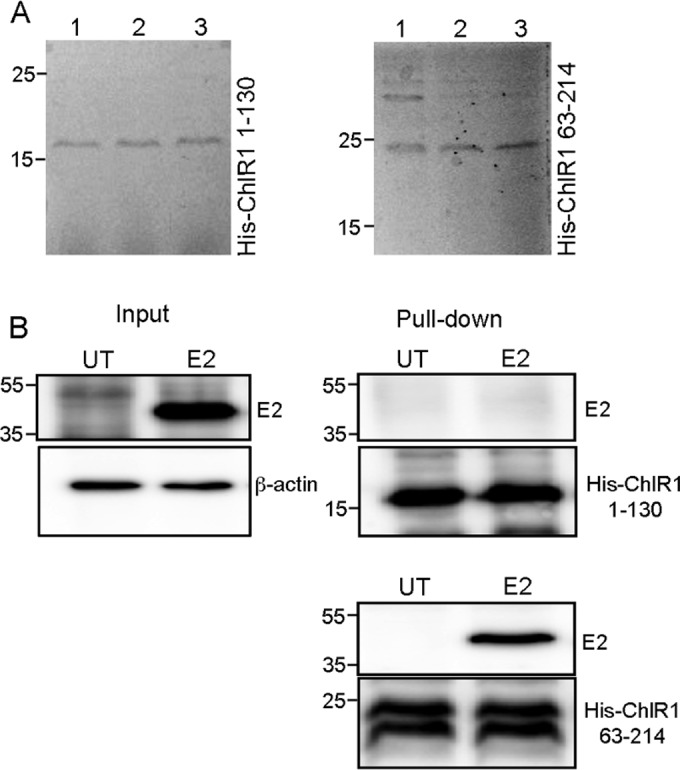
HPV16 E2 associates with the N terminus of ChlR1. (A) The His-tagged ChlR1 1-130 and 63-214 peptides were expressed and purified from E. coli and then eluted from nickel affinity resin in fractions designated 1, 2, and 3. (B) Western analysis of His-ChlR1 pulldown assays of E2 expressed in C33a cell lysates (input shown on the left). E2 associated with His-ChlR1 63-214 but not with His-ChlR1 1-130. Molecular mass markers (in kilodaltons) are indicated to the left of each panel. UT, untransfected.

Following on from our previous experiments showing that BPV1 E2 associates with ChlR1 and that mutation of tryptophan 130 to arginine (W130R) abrogates ChlR1 binding (17), we aimed to determine whether this interaction surface is conserved in the HPV16 E2 protein. To do this, we established an *in vitro* binding assay in which two N-terminal fragments of ChlR1 were cloned and expressed as hexahistidine-tagged fusion proteins. Since an N-terminal portion of Saccharomyces cerevisiae Chl1 between amino acids 190 and 280 was identified as binding to BPV1 E2 in a yeast two-hybrid screen (17), we predicted that the E2 binding region within human ChlR1 would also exist within the N terminus of ChlR1. We therefore expressed and purified amino acids 1 to 130 and 63 to 214 of ChlR1 (His-ChlR1 1-130 and His-ChlR1 63-214, respectively) (Fig. 1A) and assessed HPV16 E2 binding following incubation of immobilized ChlR1 peptides with a whole-cell lysate of HPV16 E2-transfected C33a cells. While HPV16 E2 bound robustly to His-ChlR1 63-214, the E2 protein did not bind to His-ChlR1 1-130 (Fig. 1B). This provides evidence that the E2 binding site within ChlR1 exists between amino acids 130 and 214 and that HPV16 E2 targets a domain of ChlR1 similar to that targeted by the BPV1 E2 protein.

**FIG 2:**
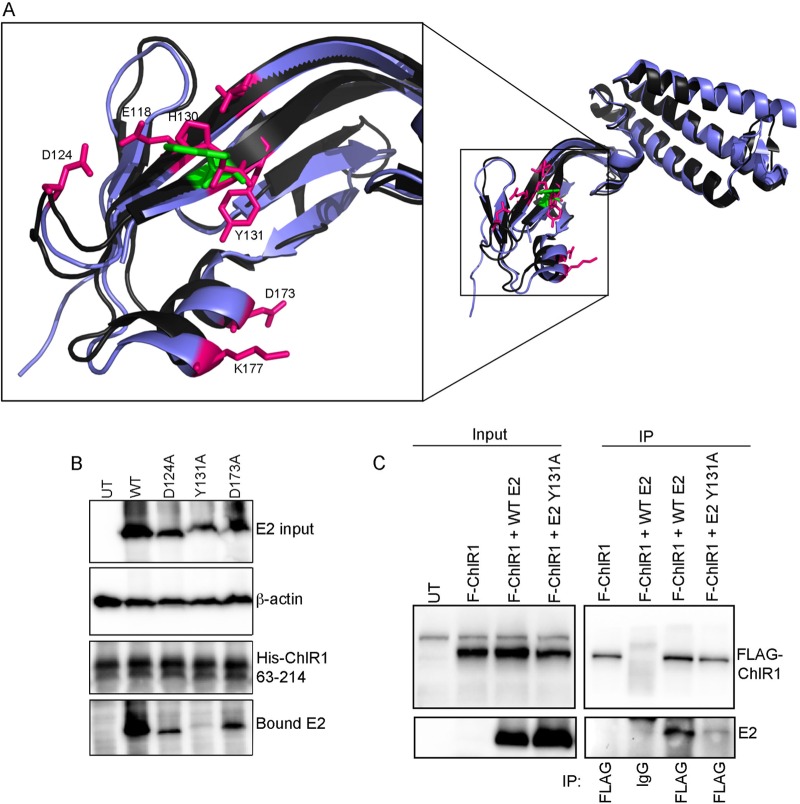
Isolation of a ChlR1-binding-defective mutant of HPV16 E2. (A) Overlay of the N-terminal transactivation domains of BPV1 E2 (black) and HPV16 E2 (purple) (produced in PyMOL). Residue W130 in BPV1 E2 is highlighted in green. The pink residues shown are the surface-exposed residues situated on the same surface of HPV16 E2 as W130 in BPV1 E2 that were mutated to alanine or arginine as described in Table 1. (B) Binding of HPV16 E2 mutants to His-ChlR1 63-214 in comparison to that of E2^WT^. Data from a representative experiment are shown, and a summary of all results is given in Table 1. (C) C33a cells growing in 10-cm dishes were transfected with 2 μg FLAG-ChlR1 and the E2^WT^ or E2^Y131A^ expression plasmid. Inputs for coimmunoprecipitation reactions are shown in the left panels. Lysates were immunoprecipitated (IP) with FLAG or nonspecific IgG, as indicated (right panels). Images are representative of three independent and consistent repeats.

To identify the amino acid residues in HPV16 E2 important for association with ChlR1, we overlaid the crystal structures of the BPV1 and HPV16 E2 proteins (19, 20) and identified amino acids within HPV16 E2 that are in close physical proximity to BPV1 E2 W130 (17) and that are surface exposed. Amino acids in HPV16 E2 (henceforth termed E2) that fit these criteria, including glutamic acid 118 (E118), aspartic acid 124 (D124), tyrosine 131 (Y131), aspartic acid 173 (D173), and lysine 177 (K177), were mutated to alanine residues. In addition, histidine 130 (H130) was mutated to an arginine, as this residue aligns with BPV1 W130, which was previously mutated to an arginine to abrogate ChlR1 binding (17) (Fig. 2A). Mutations were confirmed by sequencing, and the effects on ChlR1 binding were determined using the *in vitro* pulldown assay described above (Fig. 2B and Table 1). With the exception of E2^E118A^, which bound ChlR1 at levels similar to those for wild-type E2 (E2^WT^), most of the point mutations created on the ChlR1 binding surface of E2 reduced binding to ChlR1 in comparison to that with the E2^WT^ protein. Notably, the E2^Y131A^ mutant was severely impaired in the ability to bind ChlR1. On average, the E2^Y131A^ protein had an 18-fold reduction in ChlR1 binding in comparison to the E2^WT^ protein between multiple experiments, and this reduction in binding was consistent.

**TABLE 1:** Analysis of ChlR1 binding of HPV16 E2 mutants in comparison to E2^WT^

Mutation	% E2 bound^*a*^
E118A	106.69
D124A	38.78
H130R	45.23
Y131A	5.43
D173A	80.51
K177A	36.34

a C33a cells were transfected with an E2^WT^ or mutant E2 expression plasmid, and cell lysates were incubated with the His-ChlR1 63-214 protein immobilized on nickel resin as described in the legend to Fig. 2. The amount of bound E2 protein was determined by Western blotting and compared to the input by densitometric analysis of digital images. The percentage of E2 mutant protein bound to His-ChlR1 63-214 was calculated in comparison to the E2^WT^ binding level. The data shown are means for at least two independent experiments.

To determine whether E2^Y131A^ was also unable to bind to ChlR1 in cells, coimmunoprecipitation experiments were performed. C33a cells were cotransfected with FLAG-ChlR1 and E2^WT^ or E2^Y131A^ expression plasmids, and lysates were immunoprecipitated with a FLAG-specific antibody or nonspecific IgG control antiserum. As expected, E2^WT^ was robustly coimmunoprecipitated with FLAG-ChlR1, but E2^Y131A^ showed severely reduced ChlR1 binding (Fig. 2C). Together, these data show that HPV16 E2 associates with ChlR1 and that tyrosine 131 is essential for efficient ChlR1 binding.

### Mutation of Y131 within the E2 transactivation domain does not affect the transcriptional activation or origin-dependent replication function of E2.

**FIG 3:**
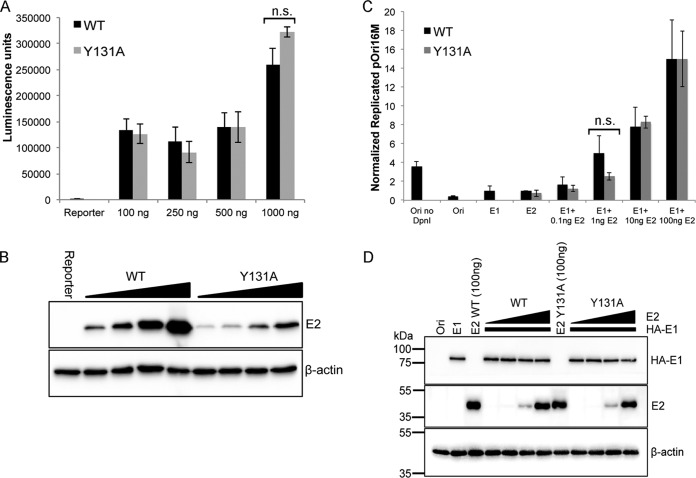
HPV16 E2^Y131A^ is transcription and replication competent. (A) C33a cells were transfected with an E2-dependent luciferase reporter (p6E2-tk-Luc) alone or in combination with increasing amounts of an E2^WT^- or E2^Y131A^-expressing plasmid. Data show mean luciferase activities and standard errors for three independent experiments. (B) E2 protein expression in comparison to that of a β-actin loading control was determined by Western blotting. (C) C33a cells were transfected with pOri16M, which contains the HPV16 origin of replication, alone or in combination with expression plasmids for HPV16 E1 only, E2 only, or a combination of E1 (constant amount) and E2 (0.1, 1, 10, and 100 ng). Replication was assessed by real-time PCR analysis of DpnI-digested DNA. Experiments were performed in triplicate, and data show the means and standard deviations for intraexperimental repeats and are representative of three independent repetitions. n.s., not significant. (D) HPV16 HA-E1 and E2 protein expression was determined by Western blotting and compared to that of a β-actin loading control.

The E2 protein is an important regulator of HPV early gene transcription and recruits the viral E1 helicase to the origin of replication (*Ori*) to initiate virus replication. These functions of E2 are essential for the virus life cycle. To determine whether E2^Y131A^ is able to support these viral functions and therefore whether interaction with ChlR1 plays a role in E2-dependent virus transcription and replication, we first utilized a synthetic transcription reporter that has previously been shown to be responsive to E2 expression in a dose-dependent manner (16). Cells were cotransfected with increasing amounts of the E2^WT^- or E2^Y131A^-expressing plasmid. Luciferase activity and corresponding E2 protein expression were determined by use of whole-cell lysates (Fig. 3A and B). E2^Y131A^ consistently activated transcription to levels similar to those with E2^WT^, demonstrating that mutation of Y131 and loss of ChlR1 interaction did not affect the transcriptional activation function of the E2 protein.

To determine whether E2^Y131A^ is able to activate virus replication to levels comparable to those with E2^WT^, cells were transfected with plasmids containing the HPV16 origin of replication (pOri16M) or expressing hemagglutinin (HA)-tagged HPV16 E1 or increasing amounts of either the E2^WT^ or E2^Y131A^ protein. Cotransfection of cells with E2^WT^ or E2^Y131A^ and HA-E1 resulted in an E2 dose-dependent activation of *Ori*-dependent replication above that observed with either *Ori* alone or *Ori* cotransfected with either HA-E1 or E2 alone. Notably, there were no significant differences in the abilities of E2^WT^ and E2^Y131A^ to activate origin-dependent replication, confirming that E2^Y131A^ is capable of activating virus replication and is as active as E2^WT^ in this assay (Fig. 3C and D).

### Loss of ChlR1 binding results in enhanced nuclear matrix association of E2.

**FIG 4:**
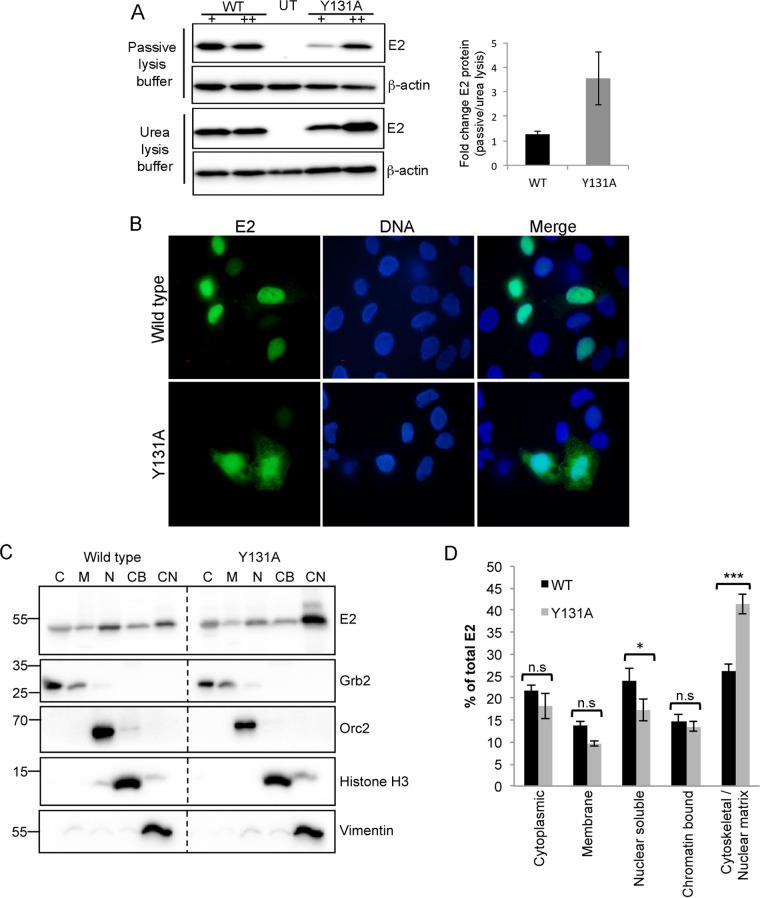
HPV16 E2^Y131A^ associates with the nuclear matrix and the cellular cytoskeleton with a higher affinity than that of E2^WT^. (A) C33a cells were transfected with 100 ng (+) or 200 ng (++) of the E2^WT^ or E2^Y131A^ expression plasmid and lysed with either passive lysis buffer, to extract soluble proteins, or urea lysis buffer, to extract all cellular proteins. Western blots for three independent repetitions were analyzed by densitometry, and the fold change in E2 protein in comparison to the loading control was calculated. (B) C33a cells growing on coverslips in 6-well dishes were transfected with 1 μg E2^WT^ or E2^Y131A^ expression plasmid, and E2 protein (green) localization was assessed by immunofluorescence staining. DNA (blue) was stained with Hoechst 33342. Bar, 10 μm. (C) C33a cells growing in 10-cm dishes were transfected with 2 μg E2 expression plasmid (E2^WT^ or E2^Y131A^) and then fractionated to give soluble cytoplasmic (C), membrane-associated (M), soluble nuclear (N), chromatin-bound (CB), and cytoskeletal/nuclear matrix-associated (CN) fractions. Fractions were analyzed by Western blotting to determine the subcellular localization of E2^WT^ and E2^Y131A^ compared to the reference proteins Grb2 (cytoplasmic and membrane-associated fractions), Orc2 (soluble nuclear fraction), histone 3 (chromatin-bound fraction), and vimentin (cytoskeletal fraction). (D) The percentage of E2 protein in each protein fraction was determined by densitometry analysis of data from three independent experimental repeats, and data shown are means and standard errors of the means. *, *P* < 0.05; ***, *P* < 0.001.

Analysis of the E2 protein expression levels in the transcription assay described above suggested that the E2^Y131A^ protein was expressed at lower levels than those of wild-type E2 (Fig. 3B). However, this reduction in E2^Y131A^ expression in comparison to that of E2^WT^ was not observed in the replication assays (Fig. 3D). This apparent difference could be explained by the different methods used to prepare cell lysates in these two experiments. The protein lysates in the transcription assays were prepared using a proprietary passive lysis buffer (Promega) which is optimized for luciferase-based assays and therefore extracts only soluble cellular proteins. In contrast, the lysates for the replication assay were prepared with urea lysis buffer, which extracts all cellular proteins, including chromatin-bound and insoluble proteins. We therefore directly compared the amounts of the E2^WT^ and E2^Y131A^ proteins extracted with these two different buffers. While the amount of E2^WT^ extracted with urea lysis buffer was similar to that extracted with passive lysis buffer, the amount of E2^Y131A^ extracted with urea lysis buffer was consistently greater than that extracted with passive lysis buffer. Over 3-fold more E2^Y131A^ was extracted with the urea buffer than with the passive lysis buffer, while no significant differences were observed for E2^WT^ (Fig. 4A). These data suggest that E2^Y131A^ is expressed at levels similar to those of E2^WT^ but is less soluble. The difference in solubility may be due to an altered association with cellular structures, such as chromatin or the nuclear matrix. Therefore, the subcellular localization of E2^Y131A^ in comparison to E2^WT^ was also determined by immunofluorescence staining. In C33a cells, the E2^WT^ protein was localized predominantly in the nuclear compartment, with some cytoplasmic staining visible, as previously reported for formaldehyde-fixed cells (18). While E2^Y131A^ was also localized predominantly in the nuclear compartment, there was a clear increase in cytoplasmic staining (Fig. 4B). This was somewhat surprising because the Y131A mutation is not within the known nuclear localization signals in E2 (21).

To analyze further the differences in subcellular localization between E2^WT^ and E2^Y131A^, transfected C33a cells were fractionated into soluble cytoplasmic, membrane-associated, soluble nuclear, chromatin-bound, and cytoskeletal/nuclear matrix fractions, and the presence of E2 in each fraction was determined by Western blotting (Fig. 4C). Quantification of these experiments revealed that there was no significant change in the level of E2^Y131A^ compared to the E2^WT^ protein in the cytoplasmic or membrane fraction. However, there was a significant reduction of E2^Y131A^ in comparison to the E2^WT^ protein (*P* < 0.05) in the soluble nuclear fraction and a concomitant increase in the proportion of E2^Y131A^ associated with the cytoskeletal and nuclear matrix fraction (*P* < 0.001) (Fig. 4D).

**FIG 5:**
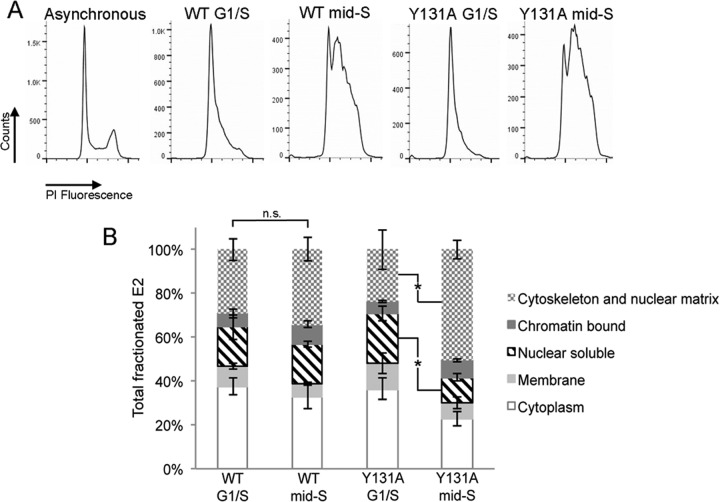
Altered subcellular localization of E2^Y131A^ in comparison to that of E2^WT^ is S phase specific. (A) C33a cells growing in 10-cm dishes were transfected with 2 μg E2^WT^ or E2^Y131A^ expression plasmid and synchronized by a double thymidine block. Cells were then either harvested (G_1_/S) or released for 3 h (mid-S phase) before staining with propidium iodide (PI). The cell cycle distribution was determined by flow cytometry of over 20,000 cells. (B) Synchronized cell populations were fractionated as described in the legend to Fig. 4, and the E2 distribution was determined by Western blotting alongside markers of the subcellular fractions. The percentage of E2 protein (E2^WT^ or E2^Y131A^) in each fraction was determined by densitometry analysis of data from three independent experiments, and the data shown are mean total E2 protein levels and standard deviations. n.s., not significant; *, *P* < 0.05.

Previous studies demonstrated that the association of BPV1 E2 with ChlR1 occurs predominantly in mid-S phase, when cellular DNA replication takes place (18). We therefore sought to determine the cell cycle dependence of the differences in E2^WT^ and E2^Y131A^ subcellular localization. E2-expressing C33a cells were synchronized at the G_1_/S boundary or in mid-S phase (Fig. 5A) and fractionated as described for Fig. 4. While the subcellular localization of E2^WT^ was not significantly altered in G_1_/S- or mid-S-phase-enriched populations of cells, there was a significant difference in the subcellular localization of E2^Y131A^ (Fig. 5B). As cells progressed to mid-S phase, the amount of nuclear soluble E2^Y131A^ was reduced 2-fold (*P* < 0.05), whereas the amount of cytoskeletal and nuclear matrix-associated E2^Y131A^ protein was increased 2-fold (*P* < 0.05), in comparison to that observed in the G_1_/S-synchronized cells. These data indicate that the interaction between E2 and ChlR1 is important for the solubility of E2 and that loss of ChlR1 binding increases the less soluble, cytoskeletal and/or nuclear matrix-associated pool of E2 protein specifically during S phase.

**FIG 6:**
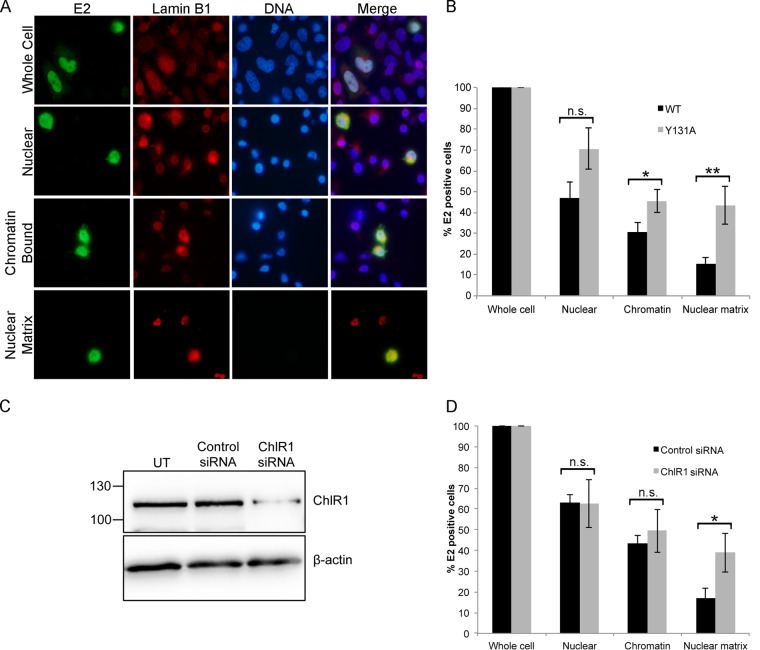
HPV16 E2^Y131A^ has reduced chromatin association and increased nuclear matrix association. (A) C33a cells growing on coverslips were transfected with 1 μg E2^WT^- or E2^Y131A^-expressing plasmid and then fixed with paraformaldehyde to observe whole-cell localization or fractionated *in situ* as described in Materials and Methods to sequentially remove cytoplasmic proteins (leaving nuclear proteins), soluble nuclear proteins (leaving chromatin-bound and nuclear matrix-associated proteins), and chromatin-bound proteins (leaving nuclear matrix-associated proteins only). The E2 protein (green) and lamin B1 protein (red) in each fraction were visualized by immunofluorescence staining, and DNA (blue) was stained with Hoechst 33342. Bar, 10 μm. (B) The percentage of E2-positive cells in each fraction in comparison to that in fixed whole cells was determined for E2^WT^- and E2^Y131A^-transfected populations. The data shown are means and standard errors for three independent repeats. *, *P* < 0.05; **, *P* < 0.01. (C) C33a cells were transfected with a ChlR1-specific or control siRNA duplex as described in Materials and Methods, and ChlR1 depletion was determined by Western blotting. (D) ChlR1-specific siRNA- and control siRNA-transfected cells were fractionated *in situ* as described for panel A, and the percentage of E2^WT^-positive cells after each fractionation step in comparison to that in fixed whole cells was determined by immunofluorescence staining. The data show means and standard deviations for three independent experiments. *, *P* < 0.05.

The fractionation experiments described thus far were performed using a biochemical fractionation method that does not distinguish between cytoskeletal and nuclear matrix-associated proteins. Therefore, to fully characterize the effect of reduced ChlR1 binding on the subcellular localization of the E2 protein and to specifically analyze the nuclear matrix attachment of E2^Y131A^ in comparison to that of E2^WT^, cells were fractionated *in situ* to visualize individual cells after sequential removal of cytoplasmic, soluble nuclear, and chromatin-bound proteins. C33a cells growing on multiple coverslips in a single tissue dish were transfected with either the E2^WT^ or E2^Y131A^ expression plasmid. *In situ* fractionation was performed, and the proportion of E2-positive cells at each fractionation step was analyzed by immunofluorescence staining of the E2 protein and normalized to the transfection efficiency calculated for the whole-cell sample. DNA and lamin B1 protein staining was also performed on each fraction to demonstrate the loss of chromatin following DNase I treatment and retention of the nuclear matrix in the final fractionation step (Fig. 6A). Removal of cytoplasmic proteins revealed no significant difference in the number of cells with nuclear E2^WT^ or E2^Y131A^ protein. However, removal of soluble nuclear proteins, leaving chromatin-bound and nuclear matrix-associated proteins, revealed a small but significant increase in the proportion of cells positive for E2^Y131A^ compared to those positive for the E2^WT^ protein (*P* < 0.05). This difference was sustained but increased in magnitude upon removal of cellular chromatin by treatment of the cells with DNase I, leaving only nuclear matrix-associated proteins. The proportion of cells that retained nuclear matrix-associated E2 was 2.8-fold greater for E2^Y131A^-transfected cells than for E2^WT^-transfected cells (*P* < 0.01). These data suggest that the ChlR1-binding-defective E2^Y131A^ protein is less tightly associated with cellular chromatin and more tightly associated with the nuclear matrix than the E2^WT^ protein.

To strengthen our conclusion that loss of ChlR1 binding results in a significant change in the solubility of nuclear E2 protein, the endogenous ChlR1 protein was depleted from E2^WT^-expressing C33a cells by RNA interference. Cells were transfected with a ChlR1-specific or nontargeting small interfering RNA (siRNA) duplex, and the reduction in ChlR1 protein expression was confirmed by Western blot analysis (Fig. 6C). *In situ* fractionation of cells was performed as described for Fig. 6A. There was no significant difference in the proportion of E2-expressing cells following removal of soluble cytoplasmic and nuclear proteins in comparison to that for control siRNA-transfected cells. However, subsequent removal of chromatin-bound proteins by DNase I treatment revealed a significant increase in the nuclear matrix association of E2^WT^ in the ChlR1-depleted cells in comparison to control siRNA-transfected cells (Fig. 6D). Together, these data indicate that disruption of the association with ChlR1, either by mutation of E2 to prevent ChlR1 binding or by siRNA-mediated depletion of ChlR1, leads to a change in solubility of the nuclear pool of E2. Loss of ChlR1 binding causes decreased chromatin association of E2, which is likely important for viral genome tethering, and increased nuclear matrix attachment.

### Reduced ChlR1 binding increases E2 protein stability.

**FIG 7:**
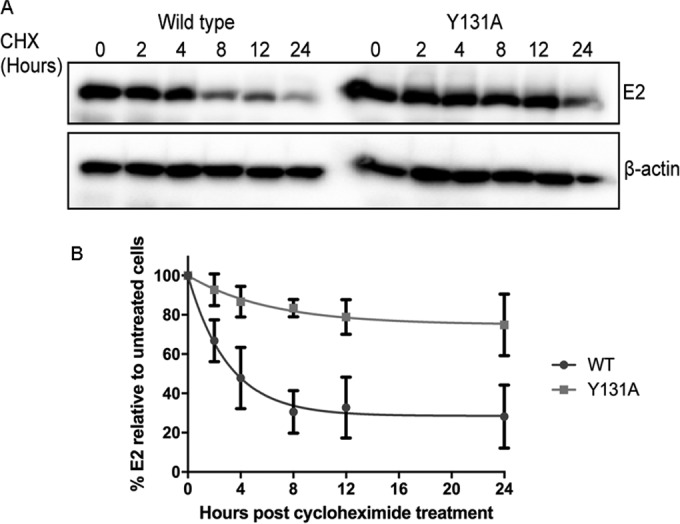
HPV16 E2^Y131A^ displays enhanced protein stability in comparison to that of E2^WT^. (A) C33a cells were transfected with 2 μg E2^WT^ or E2^Y131A^ expression plasmid. Twenty-four hours following transfection, cells were seeded into 6-well dishes and treated with cycloheximide (CHX) as described in Materials and Methods. Cells were harvested at the stated time points and lysed in urea lysis buffer, and E2 protein levels were determined by Western blotting. (B) Relative E2 protein levels were normalized to the β-actin loading control level at each time point following densitometric analysis of digitally imaged Western blots. Data show means and standard deviations for three independent experimental repeats.

It has previously been shown that overexpression of the C-terminal domain of Brd4, which contains the E2 binding domain, dramatically increases E2 protein stability by blocking ubiquitylation of E2 by the cullin-3 E3 ligase complex (22). In addition, interaction of E2 with TaxBP1, a component of the cullin-3 complex, inhibits proteasome-mediated degradation of E2 (23). This regulation of E2 protein stability is not limited to interacting cellular proteins; interactions with the E1 viral helicase and the intermediate-late protein E1^E4 also increase E2 protein stability (24, 25), although the mechanism of E1- and E1^E4-mediated E2 stabilization is not clear. In light of our data showing that ChlR1 interaction regulates the chromatin- and nuclear matrix-associated cellular pools of E2, we hypothesized that ChlR1 interaction also plays a role in regulating E2 protein stability, since attachment of E2 to cellular structures, such as the nuclear matrix, might inhibit proteasome-mediated degradation. Therefore, to determine whether the E2^Y131A^ protein has an altered stability compared to that of E2^WT^, C33a cells transfected with either E2^WT^ or E2^Y131A^ were treated with cycloheximide to inhibit protein synthesis, and the E2 protein half-life was determined by analysis of E2 protein levels by Western blotting at increasing time intervals (Fig. 7). Surprisingly, these experiments revealed a consistent and dramatic increase in the half-life of E2^Y131A^ compared to that of E2^WT^, from 4 h (E2^WT^) to 44 h (E2^Y131A^), even though the steady-state expression levels of E2^WT^ and E2^Y131A^ were comparable (Fig. 7, 0 h). This increase in protein stability without a noticeable difference in protein expression levels may have been due to the high levels of protein production in these transient-transfection assays, which may mask differences in protein stability unless the continued production of protein is inhibited by treatment with cycloheximide. Nonetheless, our data clearly show an increased stability of E2^Y131A^ in comparison to that of E2^WT^ for cells treated with cycloheximide.

### Association of HPV16 E2 with ChlR1 is required for high-copy-number viral genome establishment and episome persistence.

**FIG 8:**
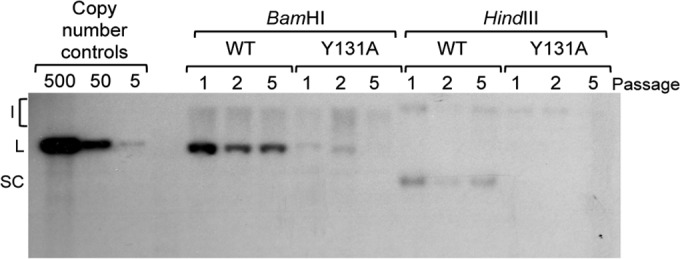
Transcription- and replication-competent HPV16 E2^Y131A^ does not support viral genome establishment and maintenance in primary human keratinocytes. Primary human foreskin keratinocytes were transfected with recircularized wild-type HPV16 genomes or HPV16 genomes encoding E2^Y131A^. Cells were harvested at passages 1, 2, and 5, and DNAs were extracted for Southern blot analysis following restriction enzyme digestion with DpnI and either HindIII, which did not cut the viral genomes, or BamHI, which linearized the viral episomes. Copy number controls equivalent to 500, 50, and 5 viral copies per cell are shown on the left. I, integrants; L, linearized; SC, supercoiled.

To determine the biological significance of the interaction between E2 and ChlR1 in the HPV16 life cycle, the Y131A mutation was introduced into the E2 open reading frame of the HPV16 genome, and recircularized viral genomes were transfected into primary human foreskin keratinocytes (HFKs) harvested from two independent donors. Following selection of transfected cells and the establishment and expansion of cell populations, total HFK DNA was harvested at subsequent early passages for analysis by Southern blotting (Fig. 8). Digestion of DNA with HindIII, a noncutter of the HPV16 genome, revealed the presence of episomes in the wild-type genome-containing cells at all three passages examined, but episomes were barely detectable in cells harboring the E2^Y131A^ mutant genome. Linearization using BamHI showed that the wild-type episomes were present at approximately 50 copies per cell at passage 1, with maintenance of episomes at a slightly lower copy number at passages 2 and 5. In the mutant genome-containing cells, the linearized genomes were present at approximately 5 copies per cells in the early passage but were not detectable at passage 5, indicating episomal loss. These observations were consistent between the two donor lines.

## DISCUSSION

Our findings provide novel insight into the role of ChlR1 in the HPV16 life cycle. We show for the first time that HPV16 E2 associates with ChlR1 and, as previously shown for BPV1 E2 (17), that the binding site for HPV16 E2 exists in the N-terminal region of ChlR1, between the highly conserved I and Ia helicase motifs. It is interesting that this region of ChlR1 is unique among related iron-sulfur cluster proteins (26), suggesting that the E2 protein evolved to specifically target ChlR1 and not related family members, such as FANCJ and XPD. Identification of a single amino acid mutation, at tyrosine 131 of E2, that severely impairs ChlR1 binding but does not affect the transactivation or replication activity of E2 allowed us to dissect the role of ChlR1 in E2 function. We show that E2^Y131A^ has a significantly enhanced half-life and is less soluble than E2^WT^, providing evidence that ChlR1 binding maintains a pool of E2 in the soluble protein fraction, which negatively affects E2 protein stability. Since functional assays demonstrate that the E2^Y131A^ mutant protein is able to support transcriptional activation and virus replication, they provide evidence that introduction of this specific mutation does not result in misfolded E2 that precipitates within the cell. We therefore conclude that the stability and solubility of E2 regulated by interaction with ChlR1 have biological importance.

Several studies have identified binding partners of E2 that regulate the stability or solubility of this essential HPV protein. Interaction with the cellular proteins Brd4 and Tax1BP1 has been shown to stabilize E2 through inhibition of the proteasome-mediated protein degradation pathway (22, 23). It has been demonstrated that interaction of HPV16 E2 with its viral binding partner, E1, increases the stability of E2 and increases the affinity of E2 for cellular chromatin (25) and that interaction with E1^E4 also increases E2 protein stability (24). Interaction with TopBP1 has been shown to regulate the solubility of E2, but, in contrast to our findings, without affecting the stability of E2 (15). As with the loss of ChlR1 binding, Donaldson et al. (15) showed that depletion of TopBP1 resulted in an increase in insoluble E2, which they suggested was due to an increase in chromatin association. However, these experiments did not specifically distinguish between chromatin-bound proteins and nuclear matrix-associated proteins, making it difficult to conclude that the less soluble fraction of E2 protein was specifically chromatin bound rather than tightly associated with the nuclear matrix. Nonetheless, the parallels between ChlR1- and TopBP1-binding-defective E2 proteins are interesting and may suggest that distinct cellular pools of E2 are bound by these proteins to regulate E2 solubility and activity at distinct stages of the cell cycle and/or virus life cycle.

The genomes of several HPV types, including HPV16, have been shown to bind with high affinity to the nuclear matrix (27). It has also been shown that the majority of the HPV11 E2 protein is tightly associated with the nuclear matrix of transfected cells and that very little cytoplasmic or chromatin-bound protein exists in these cells (28). However, the steady-state distribution of HPV16 E2 is somewhat different; our findings show that only around 30% of the cellular pool of HPV16 E2^WT^ is associated with the nuclear matrix, while the rest of the protein is distributed throughout the chromatin-bound and soluble nuclear and cytoplasmic compartments of transfected cells. Interestingly, it was shown previously that a conserved patch of basic amino acids within the hinge region of HPV11 E2 is essential for strong attachment to the nuclear matrix (28). Mutation of this patch of basic amino acids in HPV11 E2 to alanine residues resulted in an increase in cytoplasmic E2 localization. Also, there was a loss of nuclear E2 foci, which are thought to be important for HPV DNA replication. It was suggested from these studies that the nuclear matrix provides a structural scaffold for E2 compartmentalization and that attachment to the nuclear matrix is important for viral DNA replication and viral mRNA transcription. Our findings suggest that the regulation of nuclear distribution of the HPV16 E2 protein is more complex and that ChlR1 binding solubilizes a pool of E2 by reducing nuclear matrix association. However, it is important to note that the differences in subcellular localization of E2^WT^ and E2^Y131A^ overexpressed in human keratinocytes may not accurately reflect the localization of these proteins when they are expressed at low levels from the viral genome, and although interaction with ChlR1 regulates the subcellular distribution of E2, our previous studies suggest that E2 expression does not affect the gross localization of ChlR1 (17, 29). Nonetheless, loss of ChlR1 association not only increases the association of E2 with the nuclear matrix but also increases the cytoplasmic pool of E2. Whether this increase in cytoplasmic E2 is due to increased protein stability or to a loss of retention in the nucleus is not clear, but our data show that the ability of ChlR1 to regulate subcellular pools of E2 is essential for viral episome maintenance, consistent with our previous findings for BPV1.

Our previous studies showed that ChlR1 is important for sister chromatid cohesion establishment and thus supports high-fidelity chromosome segregation during mitosis (29). It has since been demonstrated that ChlR1 is important for heterochromatin organization and that its depletion causes decreased chromatin compaction at pericentric and telomeric chromosomal regions (30). Furthermore, *in vitro* assays showed that the ChlR1 helicase efficiently unwinds DNA structures that contain G-quadruplex (G4) structures (31), which are frequently found in heterochromatin (32). This ability to resolve G4 structures that inevitably stall replication forks may help to explain why ChlR1-deficient cells are highly sensitive to DNA cross-linking agents, such as cisplatin and mitomycin C, that stall the replication machinery (33). In yeast, the ChlR1 homologue, Chl1, genetically interacts with the alternative replication factor C complex protein RFC-Ctf18, which is involved in sister chromatid cohesion establishment and replication fork stabilization (34). In addition, ChlR1 is known to associate with replication fork proteins, such as proliferating cell nuclear antigen (PCNA) and the flap endonuclease, Fen1 (35, 36), and is important for replication recovery following DNA damage (37). Given these roles in replication fork stability and replication-coupled DNA damage resolution, the association between the HPV E2 protein and ChlR1 may conceivably result in recruitment of E2 to stalled replication complexes, although it should be noted that we have not demonstrated that E2 directly binds ChlR1. Nonetheless, our data show that a loss of ChlR1 binding results in reduced nuclear matrix association of E2 and in its increased solubility. Indeed, we previously showed that BPV1 E2 specifically targets ChlR1 during active DNA replication in S phase (18), which likely explains why the shift in soluble nuclear E2 to insoluble nuclear matrix-associated E2 following abrogation of ChlR1 binding is significant in cells synchronized in S phase and not in cells synchronized at the G_1_/S boundary. Put together, our data suggest that E2 targets ChlR1 during DNA replication to allow positioning of the E2-HPV genome complex at the replication fork when ChlR1 is recruited to difficult-to-replicate sites. This reduces the nuclear matrix association of the E2 protein and facilitates loading of the viral genomes onto cellular chromatin prior to mitotic segregation. This mechanism of E2-HPV genome attachment to cellular chromatin is essential for the establishment and persistence of high-copy-number HPV episomes.

## MATERIALS AND METHODS

### Plasmids.

The HPV16 E2 expression plasmid pCMV-16E2 was a kind gift from Iain Morgan, University of Virginia, and was used as a template for site-directed mutagenesis, using *Pfu* Ultra II HS DNA polymerase (Agilent), to introduce the mutations described in Table 1. pCMV-FLAG-ChlR1 expresses N-terminally FLAG-tagged ChlR1 (29) and was obtained from Jill Lahti, St. Jude Children's Research Hospital, and used as a template to clone amino acids 1 to 130 and 63 to 214 of ChlR1 into the NcoI- and EcoRI-digested pHIS-TEV bacterial expression vector (gifted by Jim Naismith, University of St. Andrews, United Kingdom [38]). The E2-dependent transcription reporter p6E2-tk-Luc encodes six E2 binding sites upstream of a thymidine kinase enhancer and carries the firefly luciferase open reading frame (gifted by Iain Morgan). For viral replication assays, plasmids p16OriM, which contains the HPV16 origin of replication, and pHPV16-HA-E1, which expresses hemagglutinin (HA)-tagged HPV16 E1 under the control of a cytomegalovirus (CMV) promoter, were also obtained from Iain Morgan (39).

The HPV16 114/K genome, cloned into pUC19, was a kind gift from Ethel-Michele de Villiers (DKFZ, Germany) and was used as a template to introduce T^3147^-to-G and A^3148^-to-C mutations to encode E2^Y131A^ by use of a QuikChange II XL kit (Agilent Technologies), the primer 5′-GATGGAGACATATGCAATACAATGCAT**GC**TACAAACTGGACACATATATAT-3′, and its reverse complement. Plasmids extracted from the resulting clones were sequenced throughout the HPV genome to ensure that no other mutations had been introduced.

### Bacterial protein expression and *in vitro* pulldown assays.

Escherichia coli BL21(DE3) was transformed with the pHISTEV-ChlR1 1-130 and pHISTEV-ChlR1 63-214 expression plasmids. Protein expression was induced by addition of 0.5 mM IPTG (isopropyl-β-d-thiogalactopyranoside) to log-phase bacterial cultures and incubation at 37°C for 4 h in an orbital shaker. Cell pellets were resuspended in lysis buffer (50 mM Na_2_PO_4_, pH 6.5, 150 mM NaCl, 1% [wt/vol] lysozyme, 5 mM dithiothreitol [DTT], and protease inhibitor cocktail) and lysed by sonication at a 35% amplitude twice for 20 s each. Lysates were cleared by centrifugation and filtration, added to a 50% Ni^2+^ resin slurry (1 ml lysate/100 μl resin), and then incubated for 2 h at 4°C with agitation. Unbound lysate was removed, and the resin was washed three times with 500 μl wash buffer (50 mM Na_2_PO_4_, pH 6.5, 150 mM NaCl, 5 mM DTT, and protease inhibitor cocktail) and resuspended in an equal volume of the same buffer. Purified proteins were assessed by SDS-PAGE and Coomassie blue staining.

### Cell culture.

C33a cells were maintained in Dulbecco's modified Eagle's medium (DMEM) containing high glucose and l-glutamine and supplemented with 10% fetal bovine serum (FBS) (Sigma). Transfections were carried out with XtremeGene HP (Roche) at a DNA/reagent ratio of 2:1. Cells were synchronized by a double thymidine block, and synchrony was confirmed following fixation of cells in 70% ethanol before incubation with propidium iodide and RNase A and analysis by flow cytometry as previously described (18). For siRNA-mediated depletion experiments, cells were first transfected with siRNA duplexes by electroporation using an Amaxa system. A total of 4 × 10^6^ cells were resuspended in 100 μl electroporation solution (Mirus), and cells were pipetted into a 0.2-cm electroporation cuvette containing 20 μl of 20 μM ChlR1-specific siRNA targeting the 3′ UTR of ChlR1 (target sequence, 5′-AGUCACUCCUUCAGUAGAAUU-3′) or a nontargeting scramble control (siGENOME nontargeting siRNA 2; Dharmacon). Cells were electroporated using program S-005, immediately plated into a 10-cm-diameter dish, and allowed to recover overnight before transfection with E2-expressing plasmid DNA by use of XtremeGene as detailed above.

### Establishment of HPV16 genome-containing primary HFKs and Southern blotting.

The transfection of normal primary human foreskin keratinocytes (HFKs) isolated from neonatal foreskin epithelia (ethical approval number 06/Q1702/45) was performed in S. Roberts's laboratory by J. L. Parish as previously described (40). To eliminate donor-specific effects, HFKs from two independent donors were used. Following G418 drug selection, cell colonies were pooled and expanded on terminally gamma-irradiated J2-3T3 fibroblasts in E medium containing epidermal growth factor. Viral genomes were extracted from each line and sequenced to ensure appropriate establishment of wild-type and mutant genomes. Organotypic rafts were prepared and cultured for 14 days in E medium without epidermal growth factor, as previously described (40). Southern blotting was performed as previously described (41).

### *In vitro* pulldown assays.

C33a cells were seeded at a density of 2 × 10^6^ into 10-cm tissue culture dishes and transfected with 2 μg E2 expression plasmid. Cells were lysed at 24 h posttransfection in 300 μl IP lysis buffer (17, 18), and 100 μl cleared lysate was mixed with equal amounts of His-ChlR1 1-130- or His-ChlR1 63-214-bound nickel resin in a total reaction volume of 300 μl made up with IP binding buffer (17, 18). Samples were incubated for 16 h at 4°C. Unbound lysate was removed, and the resin was washed three times with 500 μl wash buffer. Bound proteins were analyzed by SDS-PAGE and Western blotting.

### Coimmunoprecipitation.

C33a cells were transfected and incubated for 24 h. Coimmunoprecipitation experiments were performed as previously described (17, 18), using a FLAG-specific antibody (M2; Sigma) or nonspecific mouse IgG (Sigma). Coimmunoprecipitated proteins were separated by SDS-PAGE and detected by Western blotting using the FLAG antibody and the E2-specific antibody TVG261 (Abcam).

### Transcription assay.

Transcription assays were performed as previously described (18, 42). C33a cells (2 × 10^5^) were seeded into each well of a six-well plate and transfected with an E2 expression plasmid (100, 250, 500, or 1,000 ng) and the luciferase reporter p6E2-tk-Luc (100 ng). Firefly luciferase activity was determined for cell lysates prepared using passive lysis buffer (Promega) following the addition of luciferase assay reagent (Promega). Protein expression was confirmed by Western blotting.

### Replication assay.

Replication assays were performed as described previously (42). Briefly, 2.5 × 10^5^ C33a cells were seeded into each well of a 6-well plate. Cells were transfected with 25 ng pOri16M and expression plasmids for HA-E1 (600 ng) alone or in combination with E2^WT^ or E2^Y131A^ (0.1 ng, 1 ng, 10 ng, or 100 ng). Forty-eight hours following transfection, cells were harvested and DNA extracted using Hirt lysis buffer (0.6% SDS, 10 mM EDTA). DNA was then purified by phenol-chloroform/isoamyl alcohol extraction followed by ethanol precipitation, and the DNA was digested with DpnI. Replicated pOri16M was quantified by real-time PCR as previously described (39). For Western blot analysis of exogenous proteins (E1 and E2), cells transfected in parallel were lysed in urea lysis buffer (50 mM Tris-HCl, pH 7.5, 8 M urea, and 14 mM β-mercaptoethanol). Protein concentrations were measured by the Bradford assay, and 25 μg protein was separated by SDS-PAGE before Western blotting.

### Subcellular fractionation.

At 48 h posttransfection, C33a cells were harvested by trypsinization and counted. Cells (5 × 10^6^) were pelleted and fractionated by sequential lysis by use of a subcellular fractionation kit (Thermo Scientific). Fractions were analyzed by Western blotting for E2 and proteins used as markers of the cellular fractions (Grb2, soluble cytoplasmic fraction; Orc2, soluble nuclear fraction; histone H3, chromatin-associated fraction; and vimentin, cytoskeletal and nuclear matrix-associated fraction).

### *In situ* fractionation.

C33a cells were seeded into plates containing multiple poly-d-lysine-coated coverslips and transfected with the HPV16 E2^WT^- or E2^Y131A^-expressing plasmid. At 48 h posttransfection, coverslips were removed and placed in 6-well plates. Cells were sequentially extracted to remove cellular fractions as previously described (43). After each step, one coverslip for each transfection was removed and fixed in 3.7% formaldehyde to allow analysis of the retained proteins by immunofluorescence staining.

### Immunofluorescence microscopy.

Formaldehyde-fixed cells were permeabilized by incubation in 0.2% Triton X-100 in phosphate-buffered saline (PBS) for 10 min (with the exception of *in situ*-fractionated cells) before blocking by incubation in 20% heat-inactivated goat serum plus 1% bovine serum albumin (BSA) in PBS for 1 h at room temperature (RT). Cells were then incubated with specific primary antibodies diluted in blocking solution for 1 h at RT before washing in PBS 3 times for 5 min each. Cells were then incubated with Alexa Fluor-conjugated secondary antibodies (Invitrogen) diluted 1:1,000 in blocking solution for 1 h at RT in the dark and then washed again in PBS 4 times for 5 min each. Hoechst 33342 (5 μg/ml) in PBS was added to the final wash to stain DNA. Stained coverslips were mounted in Fluoroshield (Sigma), and cells were visualized with a Nikon E600 epifluorescence microscope fitted with a DXM1200F digital camera.

### Protein stability assay.

Protein stability assays were carried out as previously described (27). Cells were harvested 0, 2, 4, 8, 12, and 24 h following the addition of 10 μg/ml cycloheximide (Sigma) and lysed in urea lysis buffer (50 mM Tris-HCl, pH 7.5, 8 M urea, and 14 mM β-mercaptoethanol). Relative amounts of protein were quantified using a Fusion FX digital chemiluminescence detection system and software, and protein half-lives were calculated with GraphPad Prism 4 software, using a one-phase exponential decay model.
